# COVID-19-Fear Affects Current Safety Behavior Mediated by Neuroticism—Results of a Large Cross-Sectional Study in Germany

**DOI:** 10.3389/fpsyg.2021.671768

**Published:** 2021-08-06

**Authors:** Madeleine Fink, Alexander Bäuerle, Kira Schmidt, Nadine Rheindorf, Venja Musche, Hannah Dinse, Sheila Moradian, Benjamin Weismüller, Adam Schweda, Martin Teufel, Eva-Maria Skoda

**Affiliations:** Clinic for Psychosomatic Medicine and Psychotherapy, LVR University Hospital Essen, University of Duisburg-Essen, Essen, Germany

**Keywords:** COVID-19 pandemic, big five personality traits, adherent safety behavior, dysfunctional safety behavior, hoarding

## Abstract

**Objectives:** Although many research studies concerning changes in personality and behavior in time of COVID-19 pandemic emerged, important questions still have not been answered. This study with a large sample aimed to give insights into the impact of personality on pandemic fear and behavior by investigating the Big Five traits, COVID-19-fear, and associated behavioral changes in a large German-speaking sample.

**Methods:** About 14,048 healthy respondents (65.5% female, 34.2% male, and 0.32% other gender/gender queer; range = 18–85 years, median age 35–44 years) participated in the survey during the COVID-19 pandemic. Two scales, “adherent” safety behavior (ASB, α = 0.857) and “dysfunctional” safety behavior (DSB, α = 0.876), three items each, measured pandemic-associated behavior. The Big Five Inventory-10 (BFI-10) tested personality traits.

**Results:** While ASB correlated negatively with extraversion (rho = −0.053, ≤ 0.001), the other four traits were positively associated, with the highest association for neuroticism (rho = 0.116, ≤ 0.001), whereas neuroticism showed a positive correlation (rho = 0.142, ≤ 0.001) with DSB, extraversion (rho = −0.042, ≤ 0.001), agreeableness (rho = −0.028, ≤ 0.001), and conscientiousness (rho = −0.025, ≤ 0.001) correlated negatively with it. Regression analyses showed a small extent of the effect of personality traits. Moreover, neuroticism mediated the association between COVID-19-fear and DSB (positive-directed).

**Conclusions:** Even though our results on correlations between personality, pandemic fear, and related behavior are in line with the existing literature studies, the analyses clearly show that the impact of personality traits, including neuroticism, on pandemic behavior is very small. Rather, pandemic fear has a much larger influence on the safety behavior mediated through neuroticism. Further studies should bear in mind that personality traits can not only have influencing effects but also mediating effects.

## Introduction

One topic has dominated not only the media but also the everyday life of each of us during the last year: the COVID-19 caused by severe acute respiratory syndrome coronavirus 2 (SARS-CoV-2). COVID-19 was officially classified on March 11, 2020, as the first pandemic virus infection since H1N1 in 2009/2010 (WHO briefing on COVID-19, 2020). In Germany and other countries, people started to buy and hoard toilet paper, disinfectants, face masks, and basic foodstuffs (such as rice, flour, and milk), while the stocks of hygiene materials in hospitals have been exhausted due to theft. Many research studies focus on calls for solidarity and social distancing to face the massive impact of the virus on people all over the world (Raygoza, 2020; Ruijter et al., 2020). Differences in the current pandemic safety behavior could be identified: a more adherent behavior or recommended preventive behavior (Musche et al., 2020), such as social distancing and handwashing, and a dysfunctional behavior, e.g., hoarding and stockpiling (Garbe et al., 2020; Weismüller et al., 2020; Schweda et al., 2021). These differences between people can have various reasons (Flowers et al., 2014) reported that psychosocial determinants (e.g., cognitions and identity) and sociocultural determinants (e.g., social context and capacity) impact pandemic behavior in the context of an influenza pandemic.

Personality has been found to show a tight connection to health behavior in various areas (Booth-Kewley and Vickers, 1994; Smith, 2006; Raynor and Levine, 2009). By definition, personality traits within individuals are stable across time and have substantial cross-situational consistency (“person-situation” debate) (Goldberg, 1993; Faulkner et al., 2004) and the study suggests that this will impact the behavior of a person even in specific and pandemic situations. Most studies investigated the influence of personality factors on COVID-19 behavior and affectivity by correlative analyses based on the Big Five model, which describes the personality in five broad traits: neuroticism, extraversion, agreeableness, conscientiousness, and openness (Allik et al., 2013). Only few investigators used other instruments (Garbe et al., 2020; Somma et al., 2020), even concerning the Dark Triad traits (Nowak et al., 2020).

According to the Big Five model, individuals who score high in neuroticism are more likely than average to experience feelings of anxiety, anger, and depression and to respond poorly to environmental stress (Widiger and Costa, 2013). They also tend to likely interpret ordinary situations as threatening and may experience small frustrations as unbearable (Widiger and Costa, 2013). Extrovert people tend to be outgoing (Widiger, 2016), sociable, talkative, and adventurous (Rammsayer and Weber, 2016). People who score high in agreeableness often behave in a cooperative, insightful, altruistic, and generous way in interaction with other persons (see Rammsayer and Weber, 2016). Conscientiousness defines individuals who are reliable, tidy, persevering, disciplined, and hardworking (Rammsayer and Weber, 2016). People displaying high levels of openness are curious, indiscriminate, culturally interested, and seek for new experiences (Rammsayer and Weber, 2016).

Most notably, neuroticism was often found to play a significant role as a predictor of several mental and physical disorders (Lahey, 2009). Recent investigations on this personality trait and its influence on the COVID-19 pandemic behavior and affectivity support the previous findings. Qian and Yahara (2020) found that neuroticism negatively predicted underestimation, material sufficiency, medical sufficiency, information sufficiency, self-rated health status, the likelihood of surviving, evaluation to others, and confidence in doctors. Moreover, people with higher scores on neuroticism show higher levels of stress, anxiety, depression, the likelihood of infection, concerns regarding family and children, influence on life and work, and preventive behavior, such as covering mouth and nose when sneezing or washing hands. However, these results are in line with the study of Abdelrahman (2020): People with higher scores of neuroticism perform more social distancing, i.e., preventive behavior. However, it was found that even though neuroticism indeed correlates with anxiety, it does not predict it (Bayanfar, 2020). Interestingly, the association between conscientiousness and preventive behavior is similar to that of neuroticism. Conscientiousness positively predicted preventive behavior and epidemic consciousness (Qian and Yahara, 2020). Higher levels of conscientiousness were correlated with more preventive behavior, such as social distancing and handwashing (Abdelrahman, 2020; Carvalho et al., 2020). Additionally, conscientious people more frequently and intensely tend to shop and stockpile toilet paper (Garbe et al., 2020). However, even though conscientiousness resembles neuroticism regarding preventive behavior, it differs when it comes to anxiety and fear: In contrast to neuroticism, higher levels of conscientiousness were associated with decreased anxiety caused by COVID-19 (Bayanfar, 2020; Watson and Clark, 2020). Investigations on the personality factors agreeableness and openness yielded similar results regarding preventive behavior. Thus, openness positively predicted epidemic consciousness, preventive behavior, and medical sufficiency. Agreeable people presented higher levels of preventive behavior, material sufficiency, self-rated health status, the likelihood of surviving, evaluation to others, and confidence in doctors (Qian and Yahara, 2020). Yet, these findings are not consistent. Another study found higher levels of agreeableness being associated with less social distancing (Abdelrahman, 2020). Agreeable people tend to be less anxious (Bayanfar, 2020; Watson and Clark, 2020), whereas openness positively predicted stress (Qian and Yahara, 2020). The personality trait extraversion positively predicted preventive behavior, self-rated health status, concerns regarding family and children, and influence on work (Qian and Yahara, 2020). However, the results of another investigation are in contrast to the above, since higher levels of extraversion were not associated with the following governmental recommendations like social distancing and handwashing (Carvalho et al., 2020). Unfortunately, the association between extraversion and perceived anxiety has not yet been examined in this study.

On the whole, the Big Five were all found to be positively associated with preventive behavior. However, the effects of neuroticism and conscientiousness were the most distinct. Although high levels of both neuroticism and conscientiousness were associated with preventive behavior, neurotic people perceived more anxiety, depression, and stress, whereas conscientiousness is related to decreased mental burdens and increased confidence about health (Qian and Yahara, 2020).

The existing studies concerning personality and the COVID-19 pandemic only examined correlative relations between personality, especially neuroticism, and pandemic fear and/or behavior (e.g., Weismüller et al., 2020). Even though research study indicates that higher scores of neuroticism relate to higher levels of stress, anxiety, depression, and preventive behavior, and neuroticism indeed correlates with anxiety (Bayanfar, 2020), the type of relation between neuroticism and these concepts/outcomes/constructs has not yet been investigated. Therefore, some research questions remained unanswered: How strong is the influence between personality and the way of dealing with the pandemic? In particular, how much of the variance in pandemic behavior is explained solely by personality traits? By investigating the Big Five traits, COVID-19-related fear, and associated behavioral changes in healthy people in a very large online survey, this study aimed to give insights into the impact of personality and its extent on pandemic fear and COVID-19-related behavior by using regression and mediation analyses. Since neuroticism has been shown to correlate with both COVID-19-fear and pandemic behavior, we expect personality traits to predict pandemic behavior and that individuals with higher COVID-19-related fear will perform a more pronounced safety behavior mediated by neuroticism.

## Materials and Methods

### Procedure

Over the time course of almost 6 months (March 10, 2020–September 14, 2020), during the first COVID-19 phase in Germany a population-based survey was distributed *via* online channels, social media, and personal contacts. In this phase, governmental requirements became restrictive, individual freedom was curtailed (e.g., prohibition of large events, closing of public facilities, and directives to minimize social contacts), and restrictions were unstable. About 19,149 individuals completed the anonymous questionnaire reported in previous studies, e.g., (Bäuerle et al., 2020a,b; Teufel et al., 2020; Weismüller et al., 2020). For analysis, only mentally healthy individuals (aged 18–85 years) were included and respondents with unclear illness and psychiatric/psychological illness (self-report) were excluded, resulting in *N* = 14,048. All participants gave written informed consent (online) before participating in the online survey. This study was conducted in accordance with the Helsinki Declaration. In agreement with the respective ethics committee, no approval procedure was required as this survey was completely anonymous.

### Assessment

Sociodemographic data were assessed such as age, gender, education, marital status, occupation, residential situation, and psychological health status.

The Big Five Inventory (BFI) is a psychometric short scale, based on the Big Five model of personality. The questionnaire assesses the five principle aspects of personality (*openness, conscientiousness, extraversion, agreeableness, and neuroticism*) on a 5-point scale (1 = “strongly disagree” to 5 = “strongly agree”). In this study, the 10-item short German version (BFI-10) was used. Retest reliability of the scales amounted to *r* = 0.58 for agreeableness, *r* = 0.72 for openness, *r* = 0.74 for neuroticism, *r* = 0.77 for conscientiousness, and *r* = 0.84 for extraversion (Rammstedt and John, 2007).

COVID-19-related fear was assessed by the item: “I worry about COVID-19” (see Bäuerle et al., 2020a,b; Hetkamp et al., 2020; Musche et al., 2020; Skoda et al., 2020, 2021; Weismüller et al., 2020; Schweda et al., 2021). Answers were given on a 7-point Likert scale ranging from “1 = strongly disagree” to “7 = strongly agree.” Hence, higher scores indicate a higher COVID-19-fear.

Based on media reports on specific behavior during the pandemic phase and expert consensus, items were generated to cover general recommendations by the WHO (WHO briefing on COVID-19, 2020) including physical distancing, increased hand hygiene, and reported behavioral changes in media including stockpiling behavior [further questionnaire in Supplementary Table 1, see (Weismüller et al., 2020)]. Two scales assessing adherent safety behavior (ASB; e.g., item “*I increasingly avoid public places/events.”*) (*M* = 4.759, *SD* = 1.879) with Cronbach's alpha of 0.857 and dysfunctional safety behavior (DSB; e.g., item “*I have bought larger quantities of basic food (flour, sugar, noodles, rice, and canned food) or will buy more in the near future.”)* (*M* = 2.526, *SD* = 1.468) with Cronbach's alpha of.876, each with three items, have been established based on the rotated component analysis by Varimax (Supplementary Table 2). Answers were given on a 7-point Likert scale ranging from “1 = strongly disagree” to “7 = strongly agree.” Items with a scale correlation lower than.30 were excluded. The items of the two subscales and the corrected item-scale correlations can be found in Supplementary Table 2. The two scales were correlated (rho = 0.356, *p* ≤ 0.001).

### Statistical Analyses

Statistical analyses were carried out using the *Statistical Program for Social Sciences SPSS* version 26 (IBM, New York). The level of significance for all analyses was set at α = 0.05. In the presence of non-normal distributions, we generated *Spearman's rho* (two-sided). We constructed a multiple regression model with the Big Five traits predicting the corona-specific behavior of the subjects. For the mediator effects, the *PROCESS Procedure for SPSS version 3.4.1* by Andrew F. Hayes (The PROCESS Macro for SPSS, SAS and R, 2020) was used (Model 4). We controlled the direct and indirect mediational effects with the bootstrapping method (see Hayes, 2018). The level of confidence for all CIs was 95%, and numbers of bootstrap samples for percentile bootstrap CIs were set at 5,000.

## Results

### Participants

In this study, 14,048 mentally healthy (no self-reported mental illness) individuals were included with ages between 18 and 85 years and a median age category of 35–44. Demographics are shown in Table 1. Most of the participants were married and were with a high school diploma or university degree. Descriptive statistics are shown in Table 2.

**Table 1:** Demographics.

	***N***	**Percent (%)**
**Gender**		
Female	9,203	65.5
Male	4,806	34.2
Other/gender queer	38	0.3
**Age category (years)**		
18–24	1,797	12.8
25–34	3,419	24.3
35–44	3,343	23.8
45–54	2,755	19.6
55–64	1,990	14.2
65–74	645	4.6
75–84	99	0.7
**Marital state**		
Single	3,896	27.7
Married	6,210	44.2
In a relationship	2,903	20.7
Divorced/separated	771	5.5
Widowed	189	1.3
Others	79	0.6
**Education**		
University degree	6,278	44.7
High school diploma	4,476	31.9
Secondary school degree (“*Realschule*”)	2,448	17.4
Secondary school degree (“*Hauptschule*”)	591	4.2
No secondary school degree	33	0.2
Other form of education	222	1.6

*N = 14,048*.

**Table 2:** Descriptive statistics.

**Predictor**	***M***	***SD***	***S (SE = 0.021)***	***K (SE = 0.041)***
Extraversion	3.381	0.964	−0.281	−0.689
Openness	3.545	0.947	−0.304	−0.588
Neuroticism	2.698	0.885	0.218	−0.549
Agreeableness	3.166	0.748	−0.155	−0.249
Conscientiousness	3.775	0.771	−0.313	0.021
COVID-19-related fear	4.18	1.901	−0.278	−1.115
Adherent safety behavior	4.759	1.879	−0.547	−0.887
Dysfunctional safety behavior	2.526	1.468	0.814	−0.190

### Correlations

COVID-19-fear showed a significant correlation with ASB (rho = 0.538) and DSB (rho = 0.383). The correlations of ASB and DSB with the Big Five traits and COVID-19-fear are shown in Table 3.

**Table 3:** Spearman's rho correlation between the scales of adherent safety behavior (ASB), dysfunctional safety behavior (DSB), and COVID-19-fear and personality traits.

	**Extraversion**	**Openness**	**Neuroticism**	**Agreeableness**	**Conscientiousness**
Adherent safety behavior (ASB)	−0.053	0.067	0.116	0.036	0.037
*P*	≤ 0.001	≤ 0.001	≤ 0.001	≤ 0.001	≤ 0.001
Dysfunctional safety behavior (DSB)	−0.042	0.033	0.142	−0.028	−0.025
*P*	≤ 0.001	0.201	≤ 0.001	≤ 0.01	≤ 0.01
COVID-19-fear	−0.046	0.050	0.238	0.042	0.002
*P*	≤ 0.001	≤ 0.001	≤ 0.001	≤ 0.001	0.851

*N = 14,048. Two-sided*.

All correlations were significant, except for DSB and openness, and COVID-19-fear and conscientiousness. Of all the correlations, the one between neuroticism and COVID-19-fear showed the highest effect, followed by the correlation between neuroticism, DSB, and ASB. Correlations between agreeableness, extraversion, and COVID-19-fear can be considered small.

### Regression Analyses

The results of multiple regression models with the Big Five traits predicting the safety behavior of the subject are shown below. With exception of extraversion, all Big Five traits predict the ASB positively, and all predictions are with a level of significance of ≤ 0.001. This model provides an explained variance of 2.6% (Table 4). DSB is only predicted by neuroticism and openness, with an explained variance of 1.9% (Table 5). COVID-19-fear was predicted by all personality traits except extraversion, with the highest variance resolution of 6.7% (Table 6).

**Table 4:** Regression analysis to predict the ASB by the Big Five traits.

**Predictor**	**β**	***βse***	***t***	***p***
Intercept	3.222		24.002	≤ 0.001
Extraversion	−0.120	−0.061	−7.022	≤ 0.001
Openness	0.137	0.069	8.149	≤ 0.001
Neuroticism	0.261	0.123	14.336	≤ 0.001
Agreeableness	0.123	0.049	5.827	≤ 0.001
Conscientiousness	0.096	0.039	4.624	≤ 0.001

*Total R^2^ = 0.026 [F(5) = 75.988; p ≤ 0.001; N = 14,048]*.

**Table 5:** Regression analysis to predict the DSB by the Big Five traits.

**Predictor**	**β**	***βse***	***t***	***p***
Intercept	1.843		17.520	≤ 0.001
Extraversion	−0.010	−0.006	−0.732	0.464
Openness	0.063	0.041	4.797	≤ 0.001
Neuroticism	0.217	0.131	15.190	≤ 0.001
Agreeableness	−0.044	−0.023	−2.672	0.008
Conscientiousness	0.012	0.007	0.762	0.446

*Total R^2^ = 0.019 [F(5) = 55.253; p ≤ 0.001; N = 14,048]*.

**Table 6:** Regression analysis to predict the COVID-19-fear by the Big Five traits.

**Predictor**	**β**	***βse***	***t***	***p***
Intercept	1.739		13.081	≤ 0.001
Extraversion	−0.032	−0.016	−1.909	0.056
Openness	0.111	0.055	6.698	≤ 0.001
Neuroticism	0.534	0.248	29.637	≤ 0.001
Agreeableness	0.173	0.068	8.290	≤ 0.001
Conscientiousness	0.044	0.018	2.145	0.032

*Total R^2^ = 0.067 [F(5) = 202.210; p ≤ 0.001; N = 10,048]*.

### Mediator Analyses

**Figure 1:**
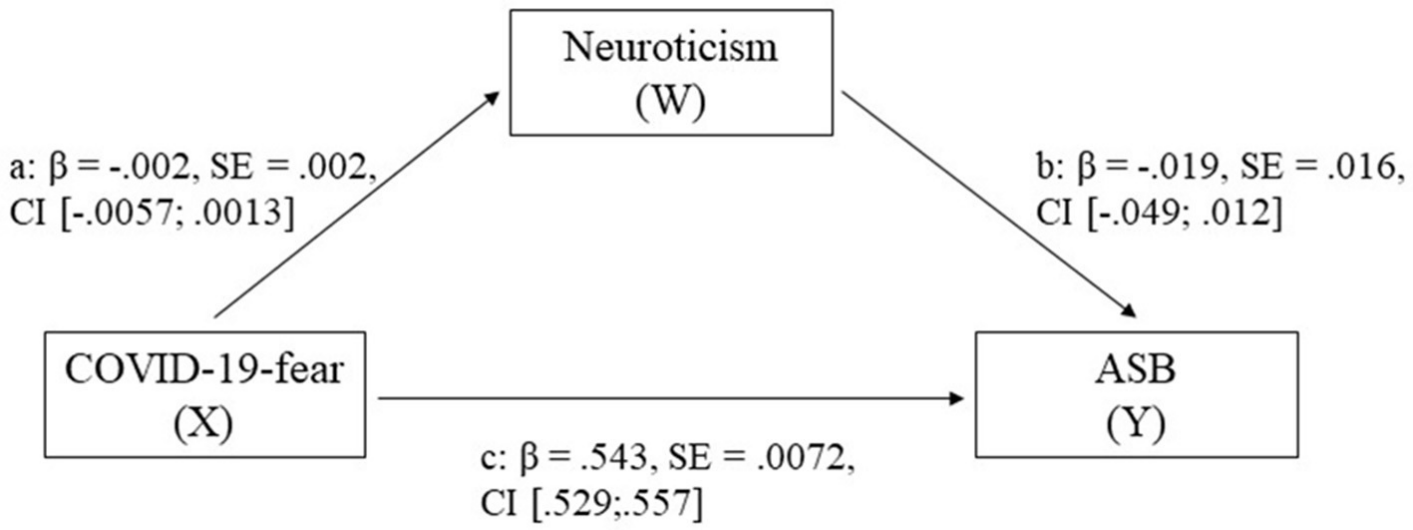
Mediation model of neuroticism on the correlation between COVID-19-fear and adherent safety behavior (ASB). c shows the direct effect of COVID-19-fear and ASB, and a and b show the completely standardized indirect nonsignificant effects by the personality trait neuroticism.

According to this study and the findings of the correlation and regression analyses (shown above), a mediation effect was assumed for neuroticism showing the highest impact on fear and behavior. The results of the mediation model of the impact of neuroticism on the relationship between COVID-19-fear and ASB for completely standardized effects and bootstrap estimates are shown in Figure 1 and Supplementary Table 3 and total *R*^*2*^ = 0.300 [*F*(2, 14,045) = 3004.923, *p* >0.001, *N* = 14,048]. The indirect effects showed now significance after bootstrapping.

**Figure 2:**
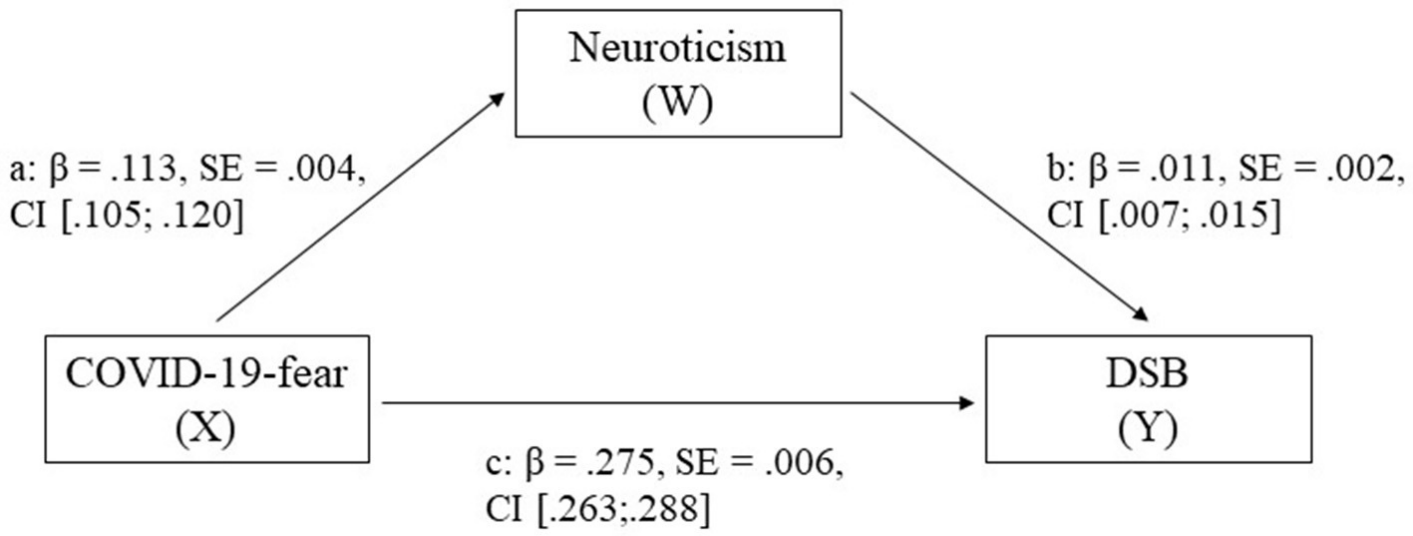
Mediation model of neuroticism on the relationship of COVID-19-fear and dysfunctional safety behavior (DSB). c shows the direct effect of COVID-19-fear and DSB, and a and b show the completely standardized indirect significant effects by the personality trait neuroticism.

The results of the mediation model of neuroticism on the relationship between COVID-19-fear and DSB for standardized effects and bootstrap estimates are shown in Figure 2 and Supplementary Table 5. All effects, within the completely standardized indirect effect of neuroticism, were significant with a total *R*^*2*^ = 0.137 [*F*(2; 14,045) = 1113.263, *p* > 0.001, *N* = 14,048].

## Discussion

Since previous studies only focused on correlations between personality and pandemic behavior, the aim of this study with one of the largest samples investigating mental health and behavior during the pandemic (*N* = 14,048) was to give a more detailed insight into the impact of personality and its extent on COVID-19-fear and pandemic-related behavior by using further analyses. Two types of behavior were detectable in this pandemic phase: a rather adherent versus a dysfunctional type of behavior. At this, the COVID-19-fear showed a significant correlation with ASB and DSB. Neuroticism was correlated with ASB, DSB, and COVID-19-fear. The regression analyses only showed a small impact of the Big Five traits on pandemic safety behavior, with an explained variance of <3%. Both the ASB and the DSB could be more effectively explained by the COVID-19-fear. Based on the previous studies on the impact of personality on current pandemic behavior (Abdelrahman, 2020; Bayanfar, 2020; Weismüller et al., 2020), mediational effects were assumed. Current analyses in this large sample showed neuroticism being understood as a mediator of the effect of COVID-19-fear on the association with DSB. There was no mediational effect on ASB. The mediation of neuroticism on fear-related DSB shows a positive direction: Individuals with a high level of COVID-19-related fear will tend to show more dysfunctional behavior mediated by their neurotic personality. Even though neuroticism correlated with COVID-19-related anxiety and also was a predictor of ASBs, no mediator effect was found for this relationship.

The previous study underlines the current correlative results for the Big Five trait neuroticism: People with a higher level of neuroticism perform more preventive behavior and are more afraid of the pandemic situation (Abdelrahman, 2020). Analyses showed clear effects of neuroticism on ASB and on hoarding or stockpiling behavior. Similar to the investigation by Carvalho et al. (2020), higher levels of extraversion were associated with less social distancing. Additionally, this study expands previous findings and shows that extroverted people show less dysfunctional behavior, e.g., hoarding. Moreover, extroverted people show less pandemic fear as well. Higher levels of the other personality traits are associated with higher expression of COVID-19-fear. According to the existing studies, openness is positively associated with adherent behavior not with dysfunctional behavior. In this study, it was found that persons who are more agreeable tend to hoard fewer disinfectants, face masks, and basic foodstuffs. According to the study by Abdelrahman (2020), it could not be demonstrated that agreeable people are less likely to practice social distancing, i.e., one of the governmental restrictions.

Looking beyond the correlations, personality effects on behavior appear extremely limited. Although neuroticism has no effect as a mediator on ASB, e.g., social distancing, it has been shown that neuroticism has a positively directed mediator effect on the relationship between COVID-19-related fear and dysfunctional behavior.

A limitation of this study is that the results can only be generalized to a certain degree, despite the immense number of cases. The current survey is based on 65.5% female, 34.2% male, and 0.3% gender queer responders. In Germany, however, gender distribution of 50.7% women and 49.3% of men has to be assumed (Statista, 2020b). Additionally, it is known that the Big Five traits depend on age (Soto et al., 2011). The current age distribution is not identical to the German population, in which the biggest cohort, with 23.5%, is built by the age-group of 40–59 years (Statista, 2020a). However, the mean age category of the current investigation was between 45 and 54 years.

## Conclusions

Nevertheless, particular strengths of this study are the information at the early stage of the pandemic and its large sample size of more than 14,000 German-speaking respondents. Already published results on the correlations between personality traits and pandemic fear and the related behavior could be replicated. However, the analyses going beyond these correlations clearly show that the extent of the effect of personality traits, even of neuroticism, can be considered very small. This study was able to show that pandemic fear has an influence on current safety behavior through neuroticism. In contrast to common assumptions by the previous studies, the analyses show high levels of neuroticism do not *per se* lead to higher anxiety but that the relationship is more complex since personality traits, in our case neuroticism, often have a mediating effect rather than just a direct effect. Therefore, greater scope for intervention can be assumed. This association should be taken into account when it comes to developing new intervention programs (see Barlow et al., 2014) and conducting governmental efforts.

## Data Availability Statement

The raw data supporting the conclusions of this article will be made available by the authors, without undue reservation.

## Ethics Statement

Ethical review and approval was not required for the study on human participants in accordance with the local legislation and institutional requirements. The patients/participants provided their written informed consent to participate in this study.

## Author Contributions

MF: conceptualization, methodology, analysis, and writing or original draft preparation. AB: conceptualization, reviewing, and editing the manuscript. MF, AB, KS, NR, VM, HD, SM, BW, AS, MT, and E-MS: reviewing and editing the manuscript. MF: visualization. MT and E-MS: supervision and project administration. All authors have read and agreed to the published version of the manuscript.

## Conflict of Interest

The authors declare that the research was conducted in the absence of any commercial or financial relationships that could be construed as a potential conflict of interest.

## Publisher's Note

All claims expressed in this article are solely those of the authors and do not necessarily represent those of their affiliated organizations, or those of the publisher, the editors and the reviewers. Any product that may be evaluated in this article, or claim that may be made by its manufacturer, is not guaranteed or endorsed by the publisher.
