# Supplementary material for: COVID-19-Fear Affects Current Safety Behavior Mediated by Neuroticism—Results of a Large Cross-Sectional Study in Germany
Source: Front Psychol. 2021 Aug 6;12:671768. doi: 10.3389/fpsyg.2021.671768 (PMC8377249; doi:10.3389/fpsyg.2021.671768)
Supplement: Supplementary file 1 [file Data_Sheet_1.docx]

**Supplementary Material**

Table I

Further Questionnaire

| Since the outbreak/spread of COVID-19 (Corona virus) in Europe... |
| --- |
| 1. *… I wash and disinfect my hands more often.* |
| 1. *… I have bought larger quantities of basic food (flour, sugar, noodles, rice, and canned food) or will buy more in the near future.* |
| 1. *… I have bought larger quantities of hand disinfection/soap/similar or will buy more in the near future.* |
| 1. *… I have bought larger quantities of toilet/hygiene articles or will buy more in the near future.* |
| 1. *… I have purchased/will purchase astronaut/emergency food in the near future.* |
| 1. *… I increasingly avoid public places/events.* |
| 1. *… I increasingly avoid public transit (subway, tram, bus, train).* |
| 1. *… I have changed my trip/vacation plans or would change them if I had planned a vacation/trip.* |
| 1. *… I have become more selfish in my behavior.* |

Likert-scale 1 = „Stimme überhaupt nicht zu“, 7 =“Stimme völlig zu“.

Table II

*Corrected Item-Scale Correction values of the scales adherent safety behavior and dysfunctional safety behavior*

| Scale ASB | Corrected Item-Scale Correlation | Scale DSB | Corrected Item-Scale Correlation |
| --- | --- | --- | --- |
| 1. I increasingly avoid public places/ events. | 0.795 | 1. I have bought larger quantities of basic food (flour, sugar, noodles, rice, and canned food) or will buy more in the near future. | 0.712 |
| 2. I increasingly avoid public transit (subway, tram, bus, train). | 0.754 | 1. I have bought larger quantities of hand disinfection/soap/similar or will buy more in the near future. | 0.704 |
| 3. I have changed my trip/ vacation plans or would change them if I had planned a vacation/trip. | 0.649 | 1. I have bought larger quantities of toilet/hygiene articles or will buy more in the near future. | 0.770 |

*N* = 14,048. *** 1 = “strongly disagree” to 7 = “strongly agree”.

Table III

*Mediation analysis of neuroticism on the relationship of COVID-19-fear and adherent safety behavior*

| Outcome Variable | *β* | *se* | *t* | *p* | *LLCI* | *ULCI* |
| --- | --- | --- | --- | --- | --- | --- |
| Neuroticism |  |  |  |  |  |  |
| Intercept | 2.227 | .018 | 127.291 | ≤.001 | 2.193 | 2.262 |
| COVID-19-fear | .113 | .004 | 29.584 | ≤.001 | .105 | .120 |
| Adherent Safety Behavior (ASB) |  |  |  |  |  |  |
| Intercept | 2.538 | .047 | 53.962 | ≤.001 | 2.446 | 2.631 |
| COVID-19-fear by neuroticism | .543 | .007 | 75,4983 | ≤.001 | .529 | .557 |

## *Total R² = .300 (F(2, 14045) = 3004.923, p > .001, N = 14,048). All direct and indirect effects were controlled with the bootstrapping method.*

Table V

*Mediation analysis for neuroticism on the relationship between COVID-19-fear and dysfunctional safety behavior*

| Outcome Variable | *β* | *se* | *t* | *p* | *LLCI* | *ULCI* |
| --- | --- | --- | --- | --- | --- | --- |
| Neuroticism |  |  |  |  |  |  |
| Intercept | 2.227 | .017 | 127.291 | ≤.001 | 2.195 | 2.261 |
| COVID-19-fear | .113 | .004 | 29.584 | ≤.001 | .105 | .120 |
| Dysfunctional Safety Behavior (DSB) |  |  |  |  |  |  |
| Intercept | 1.174 | .041 | 28.792 | ≤.001 | 1.097 | 1.251 |
| COVID-19-fear by neuroticism | .275 | .006 | 44.121 | ≤.001 | .263 | .288 |

Total *R² = .137 (F(2; 14,045) = 1113.263, p > .001, N = 14,048). All direct and indirect effects were controlled with the bootstrapping method.*
